# All 37 Mitochondrial Genes of Aphid *Aphis craccivora* Obtained from Transcriptome Sequencing: Implications for the Evolution of Aphids

**DOI:** 10.1371/journal.pone.0157857

**Published:** 2016-06-17

**Authors:** Nan Song, Hao Zhang, Hu Li, Wanzhi Cai

**Affiliations:** 1 College of Plant Protection, Henan Agricultural University, Zhengzhou, China; 2 Henan Vocational and Technological College of Communication, Zhengzhou, China; 3 Department of Entomology, China Agricultural University, Beijing, China; Nanjing Agricultural University, CHINA

## Abstract

The availability of mitochondrial genome data for Aphididae, one of the economically important insect pest families, in public databases is limited. The advent of next generation sequencing technology provides the potential to generate mitochondrial genome data for many species timely and cost-effectively. In this report, we used transcriptome sequencing technology to determine all the 37 mitochondrial genes of the cowpea aphid, *Aphis craccivora*. This method avoids the necessity of finding suitable primers for long PCRs or primer-walking amplicons, and is proved to be effective in obtaining the whole set of mitochondrial gene data for insects with difficulty in sequencing mitochondrial genome by PCR-based strategies. Phylogenetic analyses of aphid mitochondrial genome data show clustering based on tribe level, and strongly support the monophyly of the family Aphididae. Within the monophyletic Aphidini, three samples from *Aphis* grouped together. In another major clade of Aphididae, *Pterocomma pilosum* was recovered as a potential sister-group of *Cavariella salicicola*, as part of Macrosiphini.

## Introduction

The rapid expansion of genomic resources and explosion of new genome sequencing technologies are revolutionizing the research of phylogenetics. Researchers are now able to obtain phylogenomic data by fast and cost-effective means. RNA-seq (RNA sequencing), also called whole transcriptome shotgun sequencing, is a technology that uses the capabilities of next generation sequencing to reveal a snapshot of RNA presence, which is often used to generate sequence data for structural and functional studies. These data resource are important for many fields of organelle research [1]. Simultaneously, transcriptome sequencing can provide a relatively large proportion of sampled cDNA clones corresponding to mitochondrial transcripts. Thus, it is possible to mine mitochondrial genes as a by-product of transcriptome sequencing.

The mitochondrion is a fundamental eukaryotic organelle, which has a central role in energy transduction via electron transport coupled with oxidative phosphorylation to produce ATP [2]. Mitochondrial DNA (mtDNA) encodes a limited number of RNAs and proteins essential for formation of a functional mitochondrion [3]. In general, the animal mtDNA is a small (15–20 kb) genome containing 37 genes, which can be divided into three categories: the protein-coding genes (PCGs) encode 13 protein subunits of the enzymes of oxidative phosphorylation, the two rRNAs of the mitochondrial ribosome (*rrnL and rrnS*), and the 22 tRNAs necessary for the translation of the proteins encoded by mtDNA [4]. The mtDNA exhibits some properties, like simple genetic structure, maternal inheritance, and very high genome copy numbers, that make it relatively easy obtained and widely used in the study of phylogenetics, molecular evolution, and conservation genetics [4]. Previous studies have shown that mitochondrial genomes (mitogenomes) are reliable markers for phylogeny reconstruction among diverse insect groups [5]. In particular, the mitochondrial cytochrome oxidase subunit 1 (*cox1*) gene has been used as a DNA barcode to identify species, for the purpose of controlling and monitoring insects that pose the greatest threats to crops as diverse as wheat, barley, beans and potatoes. This has further increased the rate of mitochondrial gene sequencing [6].

Compared with conventional approaches [7], transcriptome sequencing can overcome the difficulty of designing a mass of species-specific primers and long-PCR amplifications. Smith (2013) advocated that RNA-seq data are an excellent and untapped resource for investigating many aspects of organelle function and evolution, and he especially emphasized that researchers do not overlook the mitochondrial-derived sequences in the RNA-seq data [1]. Typically, the transcriptome datasets produced by RNA sequencing include all the mitochondrial PCGs and most of the rRNA genes [8]. In comparison, tRNA genes are less covered. This might be because yields of mRNA are much higher than tRNA in RNA-seq, due to the excision of endonucleases. Whereas, it is believed that this situation can be improved by greater sequencing depth [7].

The cowpea aphid, *Aphis craccivora*, is an important insect pest that can cause significant losses in cereal crops and locust trees in China. Some plant pathogenic viruses, such as soybean mosaic potyvirus (SoyMV) and peanut stripe virus (PStV), have been reported to be transmitted by this aphid [9, 10]. These transmitted viruses usually inflict more dramatic damage on crops than direct injury caused by feeding of *A*. *craccivora*. The *A*. *craccivora* is classified into the family Aphididae (Hemiptera: Aphidoidea), which comprised of approximate 5,000 known species worldwide. Most aphids have intricate life cycles, of which cyclical parthenogenesis is characteristic of these insects. Many aphid species are monophagous, while others feed on hundreds of plant species across many families. Sometimes plant host alternation is related to the polyphagous aphids. To understand the evolution of insect-plant associations and to further control aphids to reduce the damage of commercial crops, the construction of reliable insect phylogenies is of primary importance. Different systematics and phylogenetics of aphids have been proposed based on morphological characters [11, 12] and molecular sequences [13–16]. Generally, three families have been recognized to constitute the superfamily Aphidoidea: the viviparous Aphididae (that are the so-called true aphids), and the oviparous Adelgidae and Phylloxeridae (both are more properly classified as aphid-like insects, because of their no cauda or cornicles) [13, 15, 17]. In the study by Nováková et al. (2013), the monophyletic Aphidini was recovered by the aphid symbiont *Buchnera* gene sequences [16]. Within the Aphidini, the monophyly of the genus *Aphis* and the sister-group relationship between *Rhopalosiphum padi* and *Schizaphis graminum* were strongly supported [16]. However, the phylogenetic relationships within the whole Aphididae are not resolved with confidence. Compared with previous molecular studies based on partial sequence fragment [13–16], mitogenome include much more phylogenetic information. Yet fewer mitogenomic data are available for aphids. At present, only seven complete (*Cervaphis quercus*, *Sitobion avenae*, *Aphis gossypii*, *Cavariella salicicola*, *Diuraphis noxia*, *Acyrthosiphon pisum* and *Schizaphis graminum*) and three partial (*Aphis glycines*, *Daktulosphaira vitifoliae* and *Pterocomma pilosum*) mitogenomes in the Aphidoidea have been published in GenBank [18–23]. Although Wang et al. (2013) presented the first comparative analysis of mitogenomes of aphids and a phylogenetic reconstructions based on 11 mitochondrial PCGs, their taxon sample are very sparse (only six aphids included in their analysis) [19]. More recently, Wang et al. (2015) sequenced the complete mitogenome of aphid *Mindarus keteleerifoliae* [24] (however, to date, this data can’t be available in GenBank, and thus this species is not included in our study). Their phylogenetic analysis based on 13 PCG nucleotide sequences from eight aphids recovered Aphidini (included only two species) and Macrosiphini (included no *Pterocomma*) in a monophyletic Aphidinae [24]. With conventional PCR and Sanger sequencing method, it is difficult to achieve the aphid mitogenome sequences, owing to the complex secondary structure, higher A + T composition and large tandem repeat regions [19].

In this study, all thirty seven mitochondrial genes of *A*. *craccivora* were obtained from transcriptome sequencing. In addition, combined with other hemipteran mitogenome sequences, the phylogenetic position of *A*. *craccivora* within Aphididae was investigated. The present study demonstrates the effectiveness of determining the whole mitochondrial gene data from transcriptome sequencing for those species with difficulty in sequencing mitogenome by conventional PCR methodology.

## Materials and Methods

### Ethics Statement

No specific permits were required for the insect specimens collected for this study in China. These specimens were collected in the residential garden of the author. The field studies did not involve endangered or protected species. *A*. *craccivora* is a common aphid species in China and is not included in the ‘‘List of Protected Animals in China”.

### Sample collection and RNA sequencing

Samples of *A*. *craccivora* (about 300 heads) were collected from locust trees on May 2015 in Zhengzhou of China (the geospatial coordinates: 34.723°N, 113.635°E). Total RNA was extracted from 35 to 50 winged adult individuals by Trizol reagent (Invitrogen, CA, USA) following the manufacturer’s procedure. The total RNA quantity and purity were determined using a Bioanalyzer 2100. Sequencing libraries were constructed using IlluminaTruSeq™ RNA Sample Preparation Kit (Illumina, San Diego, CA, USA). The results of RNA quantity and purity checking were the following: the concentration was around 3.15 ug/ul, A260/280 was 2.09, A260/230 was 1.52, total content was 300 ug, 28S/18S was 1.1, and RIN was 8.0. Prior to sequencing, two samples were prepared for repeat checking. The best run was used for further analysis. The RNA transcript was sequenced on an Illumina (Solexa) GAII sequencing machine in Shanghai OE Biotech CO., LTD. The sequencing depth was set to 4 Gb Raw data for each sample.

### Transcripts assembly and mitochondrial gene identification

The transcripts were assembled *de novo* using the Trinity method [25], and the results were inputted into BioEdit version 7.0.5.3 [26] to build a local BLAST to search genes using published aphid mitogenomes (mainly based on the *A*. *glycines* and *A*. *gossypii*) as bait sequences. Table 1 lists the analyzed aphid species, the taxon status and the GenBank accession numbers [27–31]. The best hit sequences were retrieved from transcript data. Then, the whole retrieved sequences were aligned against all the published aphid mitochondrial sequences to identify the gene boundaries. The clover-leaf secondary structures of tRNA genes predicted by tRNAscan-SE server [32] are presented in S1 Fig. New mitochondrial DNA sequences obtained in this study were deposited in GenBank under accession number of KT889380.

**Table 1:** Taxonomic information and GenBank accession numbers for the taxa included in this study.

Higher Taxon	Superfamily	Family	Subfamily	Tribe	Species	Accession number	References
Cimicomorpha	Cercopoidea	Aphrophoridae			*Philaenus spumarius*	AY630340	[27]
	Cercopoidea	Cercopidae			*Abidama producta*	NC_015799	[28]
Fulgoromorpha	Fulgoroidea	Delphacidae			*Laodelphax striatella*	JX880068	[29]
	Fulgoroidea	Fulgoridae			*Lycorma delicatula*	EU909203	[30]
	Fulgoroidea	Flatidae			*Geisha distinctissima*	FJ230961	[31]
Sternorrhyncha	Psylloidea	Psyllidae			*Pachypsylla venusta*	NC_006157	[18]
	Aphidoidea	Aphididae	Aphidinae	Aphidini	*Schizaphis graminum*	AY531391	[18]
	Aphidoidea	Aphididae	Aphidinae	Aphidini	*Rhopalosiphum padi*	Unpublished data	This study
	Aphidoidea	Aphididae	Aphidinae	Aphidini	*Aphis craccivora*	KT889380	This study
	Aphidoidea	Aphididae	Aphidinae	Aphidini	*Aphis glycines*	KC840675	[19]
	Aphidoidea	Aphididae	Aphidinae	Aphidini	*Aphis gossypii*	NC_024581	[20]
	Aphidoidea	Aphididae	Aphidinae	Macrosiphini	*Acyrthosiphon pisum*	FJ411411	Moran et al, Unpublished
	Aphidoidea	Aphididae	Aphidinae	Macrosiphini	*Diuraphis noxia*	NC_022727	[21]
	Aphidoidea	Aphididae	Aphidinae	Macrosiphini	*Sitobion avenae*	NC_024683	[22]
	Aphidoidea	Aphididae	Aphidinae	Macrosiphini	*Cavariella salicicola*	NC_022682	[19]
	Aphidoidea	Aphididae	Pterocommatinae	Pterocommatini	*Pterocomma pilosum*	KC840676	[19]
	Aphidoidea	Greenideidae			*Cervaphis quercus*	NC_024926	[23]
	Aphidoidea	Phylloxeridae			*Daktulosphaira vitifoliae*	DQ021446	Baumann and Baumann, Unpublished

### Sequence alignment and characteristics analyses

In total, eighteen mitogenome data included twelve aphids, and comprised the ingroup (10 species from Aphididae, each one from Greenideidae and Phylloxeridae, respecitively), and six other homopteran insects, and selected as outgroup (one taxa from Psylloidea, 2 from Cercopoidea, and 3 from Fulgoroidea). Each of the 37 mitochondrial genes were aligned separately for further analyses. For PCGs, firstly stop codons were excluded. Subsequently, each was aligned based on the invertebrate mitochondrial genetic code with Perl script TransAlign [33]. Both the mitochondrial tRNA and rRNA genes were aligned with reference to the conserved secondary structure. Every tRNA gene was aligned manually. Each of the two rRNAs was aligned by the R-Coffee web server [34]. Finally, all alignments were concatenated in a single matrix using FASconCAT_v1.0 [35].

Nucleotide composition of these sequences was calculated using MEGA 6 [36]. Sequence potential saturation was assessed using the index of substitution saturation (*Iss*) of Xia et al. (2003) [37] implemented in the DAMBE 5 [38]. To detect nucleotide homogeneity across taxa, the chi-square test was performed for the concatenated datasets using PAUP*4.0b10 [39]. Estimates of nonsynonymous (*dN*) and synonymous (*dS*) substitution rates of concatenated protein-coding genes were obtained by Yang and Nielsen (2000) method [40] using the program yn00 as implemented in PAML 4.9 [41]. The software SPSS 16.0 was used to perform one-way ANOVA analyses in order to test for significant differences of substitution rates between aphid lineages. For 12 aphid species, three groups were designed as the priori independent variable. Of which five species from Aphidini made up the group 0, four from Macrosiphini made up the group 1, and the remaining three aphids made up the group 2 (Table 2). The values of *dN*, *dS and dN*/*dS* were set to be the dependent variables for each test, respectively. The post hoc multiple comparisons were conducted using the method of the Least-significant difference (LSD), and the significance level was set to be 0.05.

**Table 2:** The non-synonymous and synonymous nucleotide substitutions calculated for each taxa.

Species	Group	*dN*	*dS*	*dN*/*dS*
*Acyrthosiphon pisum*	0	0.0396	2.3374	0.0169
*Diuraphis noxia*	0	0.0447	2.8293	0.0158
*Sitobion avenae*	0	0.0405	2.4343	0.0166
*Cavariella salicicola*	0	0.0472	2.4823	0.019
*Aphis craccivora*	1	0.0408	2.3948	0.017
*Aphis gossypii*	1	0.0415	2.3349	0.0178
*Aphis glycines*	1	0.0421	2.1178	0.0199
*Rhopalosiphum padi*	1	0.0416	2.4355	0.0171
*Schizaphis graminum*	1	0.042	2.7876	0.0151
*Pterocomma pilosum*	2	0.0482	2.0899	0.0231
*Cervaphis quercus*	2	0.0831	4.6348	0.0179
*Daktulosphaira vitifoliae*	2	0.1016	4.7822	0.0212

*dN*, non-synonymous nucleotide substitutions; *dS*, synonymous nucleotide substitutions.

### Phylogenetic analyses

Tree searches were conducted on the combined dataset using both Maximum likelihood (ML) and Bayesian inference (BI). Before undertaking ML analyses, PartitionFinder was employed to infer the optimal partitioning strategy [42], meanwhile the best-fitting model was selected for each partition using the Bayesian Information Criterion (BIC). The data blocks were defined by gene types (each genes of 13 PCGs as independent blocks, while both tRNA and rRNA as two blocks) and by codon positions, in total 41 blocks were utilized. The partition schemes and best-fitting models selected are presented in S1 Table.

ML searches were carried out using the partition schemes and the selected models described above with RAxML as implemented in the CIPRES Portal [43]. Support for nodes was assessed with the fast bootstrap method using 1000 non-parametric bootstrap inferences. The impact of outgroup on the phylogeny were assessed by RAxML analyses based on the combined datasets with reduced taxa (removing partial or entire outgroups). The parameter settings are as those described above.

The BI analyses were conducted using PhyloBayes with a parallel version (pb_mpi1.5a) [44, 45] as implemented on a HP server with twenty-four CPU and 64 G memory. The GTR-CAT model was used for nucleotide analyses. Two chains were run, and started from a random topology. The Maximum “maxdiff” value to be accepted was set as 0.1.

## Results

### Mitochondrial gene sequences from transcriptome sequencing data

In total, the sequenced 13,788 nucleotides of *A*. *craccivora* contained all the 37 mitochondrial genes typically present in insect mitogenomes. Of them, eleven complete PCGs (i.e., *atp6*, *atp8*, *cox1*, *cox2*, *cox3*, *cytb*, *nad1*, *nad3*, *nad4*, *nad4l* and *nad6*) were identified. There were 341 bp nucleotides missed for 3’ end of *nad2* on major strand compared to other aphid mitochondrial genomes, and 75 bp for 5’ end of *nad5* on minor strand. Compared with closely related species (e.g., *A*. *glycines* and *A*. *gossypii*), the 3’ end of *rrnS* gene determined from transcriptome sequencing of *A*. *craccivora* lacked 118 bp sequences on the minor strand. For the *rrnL* gene, there were 24 bp or 23 bp nucleotides missed in 5’ end on minor strand compared with *A*. *glycines* or *A*. *gossypii*. For the 22tRNA genes, each had a similar gene length to the released aphid mitogenomes.

All the newly determined genes exhibited strong AT nucleotide bias, such that A + T frequencies of PCGs were 82.77%, rRNAs were 84.69% and tRNAs were 84.71%. The results of the substitution saturation tests showed that the value of substitution saturation index for the combined dataset (*Iss* = 0.4853) was significantly lower than the critical values (*Iss*.*cSym* = 0.8494 or *Iss*.*cAsym* = 0.6575). This indicated that the combined data suitable for further phylogenetic analysis. The chi-square test of homogeneity of base frequencies across taxa indicated that there was significant heterogeneity among taxa for the combined dataset (*p* < 0.05).

### Protein-coding genes

Compared with the aphid *A*. *glycines* using all assembled contigs of *A*. *craccivora*, homology between each PCG gene from two species were shown as following: *atp6* 89%, *atp8* 85%, cox1 *93*%, *cox2* 95%, *cox3* 91%, *cytb* 92%, *nad1* 95%, *nad2* 91%, *nad3* 91%, *nad4* 95%, *nad4l* 97%, and *nad6* 89%. Because the partial mitogenome of *A*. *glycines* contained no *nad5* gene, there was no blast of this one. With regard to another closely related aphid *A*. *gossypii*, homology between each PCG gene from two species were similar to comparisons between *A*. *craccivora* and *A*. *glycines* (i.e., *atp6* 91%, *atp8* 90%, *cox1* 93%, *cox2* 95%, *cox3* 92%, *cytb* 92%, *nad1* 94%, *nad2* 91%, *nad3* 92%, *nad4* 94%, *nad4l* 95%, *nad5* 94%, and *nad6* 92%). Analyses of polymorphic sites between *A*. *craccivora* and two close aphid isolates were provided in Table 3. The results showed that the polymorphic sites detected between *A*. *craccivora* and *A*. *glycines* or *A*. *gossypii* were usually more than those between *A*. *glycines* and *A*. *gossypii*. This might indicate that the relationship between *A*. *glycines* and *A*. *gossypii* was closer to *A*. *craccivora*.

**Table 3:** Analyses of polymorphic sites among *Aphis craccivora*, *Aphis glycines* and *Aphis gossypii*.

Gene regions	Total length (bp)^a^			Variable sites (bp)			Total number of Synonymous changes (bp)			Total number of Replacement changes (bp)		
	*craccivora*	*glycines*	*gossypii*	*craccivora*	*craccivora*	*glycines*	*craccivora*	*craccivora*	*glycines*	*craccivora*	*craccivora*	*glycines*
				vs *glycines*	vs *gossypii*	vs *gossypii*	vs *glycines*	vs *gossypii*	vs *gossypii*	vs *glycines*	vs *gossypii*	vs *gossypii*
***atp6***	648	648	648	68	54	42	47	41	31	21	13	11
***atp8***	153	153	153	22	16	13	11	8	9	11	8	4
***cox1***	1530	1530	1530	103	102	76	97	98	70	6	4	6
***cox2***	669	669	669	27	31	22	23	28	19	4	3	3
***cox3***	783	783	783	66	58	40	51	47	34	15	11	6
***cytb***	1113	1113	1113	79	87	49	65	75	42	14	12	7
***nad1***	927	927	927	45	51	38	32	38	30	13	13	8
***nad2***	975	975	975	57	55	71	34	29	41	23	26	30
***nad3***	351	351	351	35	30	21	21	17	15	14	13	6
***nad4***	1308	1308	1308	64	60	41	55	51	32	9	9	9
***nad4l***	288	288	288	6	12	8	6	11	7	0	1	1
***nad5***	1650		1650		94			61			33	
***nad6***	486	486	486	45	51	24	24	37	13	21	14	11

^**a**^ Total length indicates sequences without stop codons.

craccivora, Aphis craccivora; glycines, Aphis glycines; gossypii, Aphis gossypii.

Of the eleven complete PCGs, five (*atp6*, *atp8*, *nad1*, *nad3* and *nad6*) used ATT as start codons, whereas *cox1*, *cox2*, *nad4* and *nad4l* used ATA, and *cox3* and *cytb* used ATG. The partial *nad2* gene had ATT to be start codons. All the PCG genes used TAA as stop codons except for *cox1* and *nad4*. The genes *cox1* and *nad4* ended with incomplete stop codons (T or TA). Because of the missing 3’ end of *nad2* and missing 5’ end of *nad5*, there were no stop codons or start codons to be found for them.

### Transfer RNA genes

The standard 22 tRNA genes were found in the transcriptome sequencing data of *A*. *craccivora* which ranged from 62 bp (*trnD*, *trnG*, *trnS-AGN*, *trnT* and *trnV*) to 73 bp (*trnK*) in size. Of them, all tRNAs could be folded into typical cloverleaf structure except for *trnS-AGN* (S1 Fig). *trnS-AGN* lacks the dihydrouridine (DHU) arm, as in many other insect species [46].

### Phylogenetic analyses

**Fig 1:**
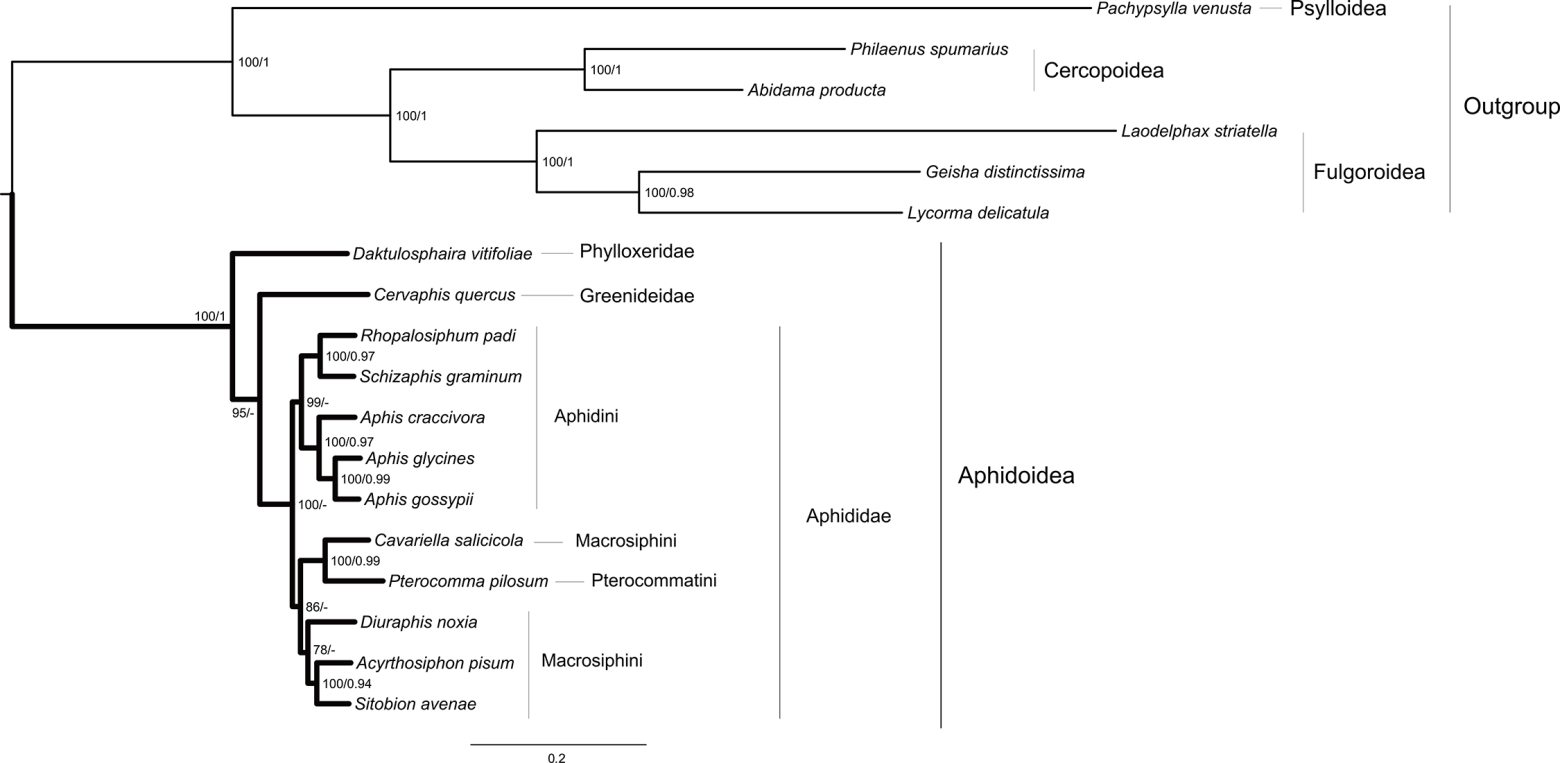
Maximum likelihood tree estimated from the combined dataset. Node numbers show bootstrap support values (above 70, left) and posterior probabilities (above 0.9, right). Scale bar represents substitutions/site.

The newly obtained full mitochondrial gene data were included in phylogenetic analyses along with other available aphid mitochondrial data. The two trees resulting from the ML and BI analyses had a similar topology (Fig 1). The only discrepancy was in the basal interfamilial relationships within Aphidoidea. ML analysis recovered the Phylloxeridae as the first branch, and the Greenideidae as the next. However, Bayesian analysis retrieved these two families as sister group with low statistical support (posterior probability 0.69). In both ML and BI trees, one notable aspect was the distinctive branch lengths seen between outgroup and ingroup taxa. In particular, outgroups *P*. *venusta* and *L*. *striatella* had longer branches in comparison to ingroup aphids. To investigate the potential effect of fast evolving outgroup on the tree topology, we successively removed the long-branched *P*. *venusta*, *L*. *striatella*, *Geisha distinctissima* and *Lycorma delicatula* or all six outgroup taxa to rerun tree searches (S2A and S2B Fig). The resulting ingroup relationships are identical to Fig 1.

Moreover, both ML and BI analyses showed strong support for a monophyletic Aphididae (BP = 100, PP < 0.9). However, within the family Aphididae, the subfamily Aphidinae was recovered as a paraphyletic group, with the Pterocommatinae (represented by *P*. *pilosum*) nested within. In addition, two tribes were strongly supported: Aphidini, and Macrosiphini including *P*. *pilosum*. Within the Aphidini, both trees strongly supported a monophyletic *Aphis*, which was found to be a sister taxon to the clade of (*R*. *padi* + *S*. *graminum*). Thus, two subtribes, Aphidina and Rhopalosiphina, were strongly supported (BP = 100, PP > 0.95). In the genus *Aphis*, the *A*. *glycines* had a closer relation to *A*. *gossypii* than to *A*. *craccivora*. This might be the fact that sequence variation (polymorphic sites) between *A*. *glycines* and *A*. *gossypii* were relatively low. For the rest of aphids, the relationships of ((*C*. *salicicola* + *P*. *pilosum*) + (*D*. *noxia* + (*A*. *pisum* + *S*. *avenae*))) were consistently supported by all analyses.

### Substitution rate analyses

The substitution rate analyses showed that the newly sequenced *A*. *craccivora* had a similar *dN* value to other aphids from the tribe Aphidini (0.0408 ~ 0.0420) (Table 2). For five aphid species from the group of the Macrosiphini (including *P*. *pilosum*), *A*. *pisum*, *D*. *noxia* and *S*. *avenae* had a relatively lower *dN* values (0.0396 ~ 0.0447), whereas *C*. *salicicola* and *P*. *pilosum* had a higher *dN* value (namely, 0.0472 and 0.0482). These results corresponded to the two clades, which was respectively constituted by the former three aphids and the latter two ones. The rest two aphids (i.e., *C*. *quercus* and *D*. *vitifoliae*) had obviously higher *dN* values than other ones. Similarly, all aphids had the *dS* values ranging from 2.0899 to 2.8293 except for the *C*. *quercus* and *D*. *vitifoliae*, and the two latters had higher *dS* values than 4.6. The statistical analyses revealed no significant differences of *dN* or *dS* values among the defined groups (P > 0.05). However, there were significant differences for *dN*/*dS* values between the group comprised by the species of Macrosiphini and the group including *P*. *pilosum*, *C*. *quercus* and *D*. *vitifoliae* (comparison between group 0 and group 2, P = 0.030), and between the group of Aphidini and the group including the *P*. *pilosum*, *C*. *quercus* and *D*. *vitifoliae* (comparison between group 1 and group 2, P = 0.036). This result basically confirmed the recovered phylogeny of aphids at the tribe level.

## Discussion

### Next generation sequencing for complete mitochondrial gene data

Traditionally, complete mitogenome data has been generated by PCR-based strategies, which can be readily confounded by degraded DNA templates, PCR amplicons and sequencing conditions, species-specific primers, and complex genome structure and organization, etc. Thus, it may be time consuming and costly. In contrast, next generation sequencing can overcome these difficulties and allows determining full mitochondrial gene data more effectively, in particular when it is becoming relatively cheaper already today. This study demonstrates the usefulness of transcriptome sequencing for obtaining complete mitogenome data. However, the obvious drawback of this approach is the inability to find out gene arrangement and to achieve the mitochondrial control region directly from transcript data. In addition, the number of mitochondrial genes detected by transcriptome sequencing varied depending on the different expression level with different sampling [8]. Sampling includes various complex factors, for example, different insect species, developmental stages, sexes and biotypes. A study of the brown planthopper *Nilaparvata lugens* revealed that long wing forms have higher expression levels of genes involved in respiration and energy metabolism compared to short wing forms [47]. They contributed this to the fact that long wing forms required more energy than short wing forms for flight. In this study, we chanced to sample the winged forms of aphid *A*. *craccivora* for transcriptome sequencing. The mitochondria is an important cell organelle responsible for energy metabolism. Thus, transcripts with abundant mitochondrial genes were obtained, and the entire set of 37 mitochondrial genes could be determined from *A*. *craccivora* transcript. This is only a speculation, the fact needs to be verified by further experiment. In general, transcriptome sequencing can provide the full set of mitochondrial PCGs and partial RNA genes, which are in fulfillment of the need of systematic research based on mitogenome sequences.

### Phylogeny

Mitogenome data are often employed for phylogenetic analyses and are useful to study the intra-order relationships of insects [48–50]. Nevertheless, attempts to reconstruct the higher mitogenomic phylogeny have often failed [51, 52]. The rapid rate of mitogenome evolution limits the resolving power of this type of molecule marker for deep phylogeny reconstruction. In the current study, our phylogenetic analyses suggest that the full mitochondrial gene data may be appropriate for providing fine resolution of evolutionary relationships for the insect family Aphididae.

Outgroup selection is important for phylogentic inference [53–58]. According to previous studies on Hemiptera phylogeny [30, 59–62], we used a comprehensive taxon sampling from other homopteran lineages which are closely related to aphids. Although some outgroup taxa exhibited obviously long-branch lengths due to higher sequence evolutionary rate, taxa-excluding analyses demonstrated that the resultant tree topology was not affected by long branches (S2A and S2B Fig). Therefore, the current outgroup choice is appropriate.

Two different inference methods under different evolutionary models resulted in congruent tree topology within the superfamily Aphidoidea (Fig 1). The families Phylloxeridae and Greenideidae were successively recovered as the early diverging lineages in Aphidoidea. This arrangement is also supported by the symbiont *Buchnera* DNA sequences [16]. The monophyletic family Aphididae and tribe Aphidini were retrieved. However, the monophyly of the subfamily Aphidinae was not supported due to the nested position of Pterocommatinae. Classifications of Aphidinae have been controversial. Until the latter half of the 20th century, most taxonomists concurred that the Aphidinae included three major groupings: “pterocommatines” (*Pterocomma*, etc.), “aphidines” (*Aphis*, *Rhopalosiphum*, *Schizaphis*, etc.), and “macrosiphines” (*Acyrthosiphon*, *Diuraphis*, *Sitobion*, etc) [14, 63]. However, the relationships among these lineages were not fully resolved. Remaudière and Remaudière (1997) grouped aphidines and macrosiphines together, both of which comprised the subfamily Aphidinae [64]. And they classified pterocommatines as the independent subfamily Pterocommatinae. But the affinities of Pterocommatinae to Aphidinae were not clear. In contrast, von Dohlen and Moran (2000) strongly supported a monophyletic group of (pterocommatines + aphidines + macrosiphines) [65]. Shaposhnikov et al. (1998) placed Pterocommatini as a sister group to the clade (Aphidini + Macrosiphini) [66]. This hypothesis was confirmed by the study of Ortiz-Rivas and Martínez-Torres (2010), based on the combined analysis of nuclear and mitochondrial sequences [15]. However, von Dohlen et al. (2006) recovered a sister-group of *Pterocomma* plus *Cavariella* [14]. And they thought that there seemed to be no morphological synapomorphies to support the grouping of (Aphidini + Macrosiphini). By contrast, they demonstrated that the distance between stigmal pores on abdominal segments could be considered as a key character to support the relationship of Pterocommatini with Macrosiphini [14]. Our mitogenomic analyses recovered the relationships of ((Pterocommatini + Macrosiphini) + Aphidini). In fact, this result had been obtained using single gene locus EF1α by Ortiz-Rivas and Martínez-Torres (2010) [15] (see Fig 2 in their paper). Another prior mitogenomic phylogeny by Wang et al. (2013) also recovered the sister relationship of *C*. *salicicola* to *P*. *pilosum*, and they advocated that pterocommatines should be transferred into Macrosiphini [19]. This point has also been confirmed by the phylogenetic analyses in the present study.

Besides *C*. *salicicola*, all the other *macrosiphines* clustered in a clade, and the sister-group of (*A*. *pisum* + *S*. *avenae*) were strongly supported (BP = 100, PP = 0.94). Close relationship between these two aphid species has been reported in previous molecular studies [14, 15]. Within the tribe Aphidini, two subtribes were recognized: Aphidina (*Aphis*, *Toxoptera*, etc.) and Rhopalosiphina (*Rhopalosiphum*, *Schizaphis*, etc.) [67]. In our analyses, this two subtribes were consistently recovered with strong nodal support (BP = 100, PP = 0.97). In addition, our data strongly support a sister group relationship between *R*. *padi* and *S*. *graminu*, as suggested by *Buchnera* DNA sequences [16]. The newly determined mitochondrial gene data of *A*. *craccivora* fell within the genus *Aphis*, and clustered with the published mitogenomes of *A*. *glycines* and *A*. *gossypii*. Thus, this result validates the approach of obtaining mitogenome sequence from transcriptome sequencing.

### Implications for aphid host alternation and pest control

For the lineage Aphidinae, the strong support found for the relationships among its tribes and subtribes, and especially about the position of *Pterocomma*, makes us to be able to discuss some issues on the evolution of life cycles and some strategies of pest control of this group under the framework of reconstructed phylogeny.

The evolution of seasonal host alternation and complex life cycle led insects from Aphidinae to be explosively diversified in Tertiary [68]. In particular, presence of winged males during the autumn migration makes the Aphidinae distinguished from other aphid lineages, and implies a separate origin in this subfamily [69]. These characteristics also enable Aphidinae to be better adapted to the modern, seasonal, north-temperate climate and vegetation [70]. Thus, investigating origins of host alternation of aphids will benefit research on aphid host range and on aphid pest control. In the present study, three aphid tribes were included in the family Aphididae: Pterocommatini, Macrosiphini, and Aphidini. The reconstructed aphid phylogeny from mitogenomic data strongly supported *P*. *pilosum* (Pterocommatini: *Pterocomma*) embedded within Macrosiphini as the sister to *C*. *salicicola*. This is congruent with the result from von Dohlen et al. (2006) [14]. Pterocommatini have simple life cycles of non-host-alternating on hosts in the Salicaceae. Whereas, both Macrosiphini and Aphidini comprised rich species with or without host-alternating life cycles on diversified host plants of Rosaceae, Asteraceae, Poaceae, etc. Based on our phylogenetic analysis, two conclusions can be drawn on the origins of host alternation of Aphididae. First, the inferred basal position of *P*. *pilosum* (Pterocommatini) in Macrosiphini from mitogenomic data is in agreement with the view that the common ancestor of Aphidinae had a simple, non-host-alternating life cycle on a woody host [14]. Second, the similar phylogenetic relationships of Aphididae found here to that from von Dohlen et al. (2006) [14] further support the idea of the existence of several independent origins of host alternating life cycles in this group of insects.

A solid phylogeny is critical for inferences not only of the evolution of host-plant associations in Aphididae, but also for pest control. For example, based on comparative genomic data, researchers can predict a drug effect on some pests when molecular targets of a compound are known or suspected [71]. The similar phylogenomic pipeline can be used to study the mechanisms behind the action of insecticides on aphid pest. Although obtaining an aphid phylogeny largely concurrent with some previous studies, this study is preliminary due to still limited taxon sampling. Future studies need to include more mitogenomes from this important agricultural insect pest group. Especially, with the explosion of new genome sequencing technologies, researchers should explore large phylogenomic data from the rapid expansion of genomic resources for mitochondrial sequences to reconstruct a reliable phylogenetic relationship of aphids.

## Supporting Information

S1 Fig The secondary structures of mitochondrial tRNAs predicted from *Aphis craccivora*. (TIF)

S2 Fig Maximum likelihood trees estimated from the combined datasets with reduced taxa. **A)** Removing long-branched outgroups: *Pachypsylla venusta*, *Laodelphax striatella*, *Geisha distinctissima* and *Lycorma delicatula*). **B)** Removing all outgroups. Node numbers show bootstrap support values (above 70). Scale bar represents substitutions/site. For S2A Fig, the internal branches between outgroup and ingroup were halved to more clearly illustrate the tree.
(EPS)

S1 Table The partition schemes and best-fitting models selected. (XLSX)
