# Supplementary figures and images for: All 37 Mitochondrial Genes of Aphid Aphis craccivora Obtained from Transcriptome Sequencing: Implications for the Evolution of Aphids
Source: PLoS One. 2016 Jun 17;11(6):e0157857. doi: 10.1371/journal.pone.0157857 (PMC4912114; doi:10.1371/journal.pone.0157857)

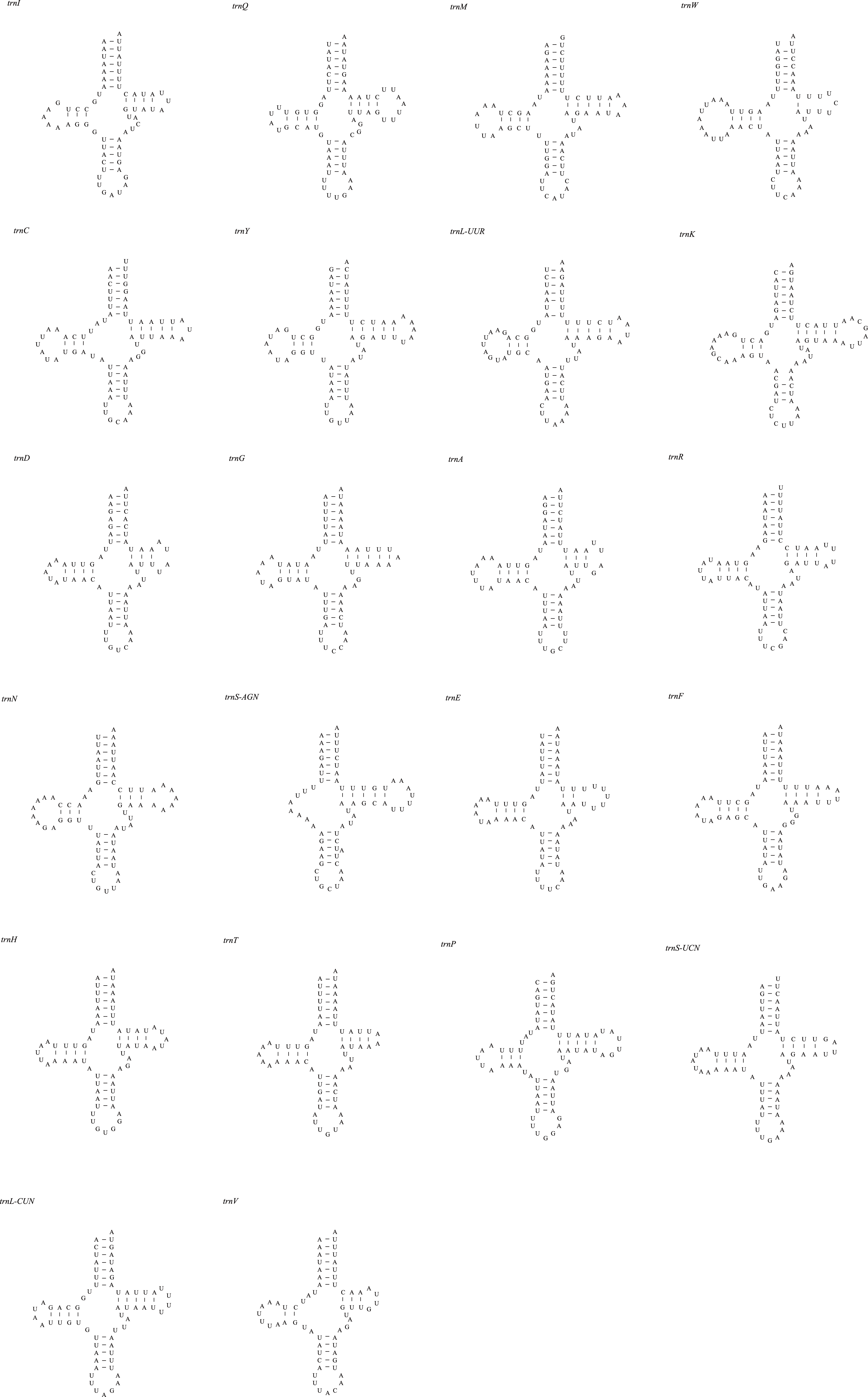

Supplement: S1 Fig — (TIF) [file pone.0157857.s001.tif]
